# Editorial: Women in neuroengineering and neurotechnologies

**DOI:** 10.3389/fnins.2025.1542839

**Published:** 2025-01-28

**Authors:** Michela Chiappalone, Alessandra Pedrocchi, Suha Abuarqub, Chiara Bartolozzi, Valentina Benfenati, Silvia Giordani, Elvira Pirondini, Marianna Semprini

**Affiliations:** ^1^Department of Informatics, Bioengineering, Robotics, System Engineering (DIBRIS), University of Genova, Genova, Italy; ^2^Nearlab, Department of Electronics Information and Bioengineering, Politecnico di Milano, Milan, Italy; ^3^We-Cobot Laboratory, Politecnico di Milano, Milan, Italy; ^4^Rockwell Automation Italia, Milano, Italy; ^5^Event-Driven Perception for Robotics, Istituto Italiano di Tecnologia (IIT), Genova, Italy; ^6^Consiglio Nazionale delle Ricerche - Istituto per la Sintesi Organica e Fotoreattività, Bologna, Italy; ^7^School of Chemical Sciences, Dublin City University, Dublin, Ireland; ^8^Department of Physical Medicine and Rehabilitation, Rehabilitation and Neural Engineering Laboratories, University of Pittsburgh, Pittsburgh, PA, United States; ^9^Rehab Technologies, Istituto Italiano di Tecnologia (IIT), Genova, Italy

**Keywords:** women, engineering, science technology engineering mathematics (STEM), neuroengineering, gender equality

When thinking about science and scientists, the first names coming to mind belong to men. That is because only a few female scientists are widely recognized for their contributions to the history of science and engineering. The reasons for this trend are well known and yet difficult to eradicate. In 1927 at the famous Solvay conference in Belgium, 29 out of 30 attendees were men. They were all prestigious scientists, such as Albert Einstein and Erwin Schrödinger. The only woman was Marie Curie, who at that time had already won two Nobel prizes. Despite the position of women in the society has changed since 1927, biases and stereotypes still remain and jeopardize a female's chances of becoming a scientist. Even if today most science departments aim at equal opportunities more than one century ago, there is still considerable progress left to make. Indeed, the gender balance is preserved mostly at early stage of career (i.e., among PhDs and young Post Docs) but in most colleges and universities, Principal Investigators are still mostly men.

Many highly influential and successful women are contributing to science and in particular to the fields of Neuroengineering and Neurotechnologies, both in the academic and non-academic sector. Yet, female scientists and managers are still underrepresented in various aspects of both academic life (e.g., keynote speakers at conference, directors of research, directors of infrastructures) and industry world (e.g., founders of tech companies, CEOs, top managers). Several initiatives have been recently created to increase the visibility of women; however, gender bias, gender gap and glass ceiling area matter of fact, if concrete actions are not taken by the politician but also by scientists, as a community and as a society.

Indeed, inclusion, equal opportunities, promotion of diversity and gender equality, is not only a goal of the United Nations 2030 Agenda, but it is essential milestone enabling the achievement of all 17 Sustainable Development Goals.

At the “IEEE Women in Engineering International Leadership Summit” held in Genova, Italy, in 2021, science, mentorship, competitiveness, leadership, innovation, diversity, and parenting have been discussed with leaders in the fields of Neuroengineering and related fields. Rooting on the results of that Summit, this Research Topic aimed at broadening the audience, actively promoting the dissemination of scientific work involving women scientists, mostly in the field of neuroscience, neural engineering, neuroprosthetics, neural and nanotechnology and computational neuroscience. Eight manuscripts were accepted and published within this Research Topic, targeting diverse article types (i.e., one Original Research, one Brief Research Report, one Review, three Opinions and two Book Reviews), but with a common denominator: promoting research and ideas driven by women.

The article by Soroushmojdehi et al. led by M. Gandolla, proposes a new methodology for decoding the hand movement intention from electromyography (EMG) signals. Since creating a large dataset for a single subject to train deep networks could be very time-consuming, the authors propose (i) a subject-transfer framework, which allows a model to use knowledge learned from other subjects' EMG data; (ii) a task-transfer framework, where the knowledge acquired from classifying basic hand movements is applied to more complex movements that involve combinations of these basic actions. Two Convolutional Neural Networks—based architectures were introduced for hand movement intention detection and a subject-transfer learning approach. Results show that the subject-transfer learning approach increased classification accuracy. Additionally, the task-transfer approach demonstrated that complex hand movements could be classified by leveraging knowledge from simpler movements. Globally, the study demonstrates that transfer learning can improve significantly the performance of EMG-based decoder for neural interface applications.

The work by Morelli et al., led by S. Signorini, introduces the TechArm system, a novel technology for visual rehabilitation. The system is capable of providing both uni- and multi-sensory stimuli (audio and tactile) to help visually impaired children to improve their ability to interpret non-visual cues. Participants (low-vision, blind, and sighted children) received auditory, tactile, or combined audio-tactile stimuli by means of four TechArm's units were placed on their forearm and were asked to identify the number of active units. Results showed that tactile stimuli led to the best performance, while auditory accuracy was near chance. The combined audio-tactile condition was more effective than auditory stimuli alone, indicating the benefits of multisensory stimulation. The reported findings support TechArm's potential in developing personalized therapies for the rehabilitation of visually impaired children.

Two Opinion articles were presented by the group of F. Tecchio. In the first one, Persichilli et al. hypothesize that the triadic principle feedback-synchrony-plasticity (FeeSyCy) governs the adaptive capacity of the body-brain system and underlies the effectiveness of Eye Movement Desensitization and Reprocessing (EMDR) to fight against major neurological disorders. In the Opinion, the authors discuss about post-traumatic stress disorder (PTSD), affecting twice women than men, as a case study, since it provides an exemplificative case of a dramatic alteration of the adaptive physiological nature of the body-brain system. In the second Opinion article, Grifoni et al. capitalize on recent findings from relevant pathophysiological contexts, to indicate therapeutic approaches in musicians' dystonia (MD), a task-specific, mostly painless, neurological condition that disrupts musicians' ability to play their instruments. The article emphasizes the necessity of diverse interventions, from sensorimotor therapies like physiotherapy to approaches targeting the subcortical areas involved in memory, identity, and emotion regulation. The adoption of this comprehensive approach will likely alleviate symptoms, build resilience, and improve quality of life for those affected by this challenging neurological condition.

Two Book reviews are also part of the Research Topic. The first one (Armonaite et al.) presents and discusses the book by A. Di Leva titled “*The fractal geometry of the brain*”. The book offers a comprehensive exploration of fractal geometry in neuroscience, presenting its applications in brain morphology, clinical analysis of neurodegenerative diseases, and computational modeling of brain dynamics, highlighting the brain's complexity through the fractal concept. The second one (Gianni and Tecchio) reviews the book by Brunoni titled “*Transcranial direct current stimulation in neuropsychiatric disorders. Clinical principles and management*”, re-issued in 2021 with a new expanded edition. The book describes the mechanisms of action of the main techniques for transcranial electrical stimulation as well as their current and potential applications, providing both perspectives for electroceutical treatments and limitations.

The last two articles published in this Research Topic are not directly related to scientific results or scientific discussions led by women. Instead, they focus on important aspects of women's life: working in challenging fields and parenthood.

The article by Jantz et al. is a well detailed review of the literature focused on the experiences of women in academic careers in fields closely related to neural engineering. They also reported interviews to women scientists, to identify many of the obstacles women face in the course of their careers and provide recommendations and materials to overcome them.

The Opinion article by Pedrocchi highlights an underappreciated perspective: parenthood as a professional asset. Instead of viewing parenthood solely through the lens of work-life balance, the author emphasizes its role in developing valuable skills such as maturity, time management, empathy, and leadership—critical in academia and STEM fields. Pedrocchi advocates for workplace policies that integrate work and family life, including flexible work arrangements and mentoring programs. Such measures encourage young professionals to embrace parenthood without fear of career setbacks, fostering a more inclusive and resilient workforce. This perspective challenges traditional narratives, emphasizing the need to recognize parenthood's contributions to both personal and professional growth while promoting a culture that values diverse life experiences.

This Research Topic gave all the authors and the Guest Editors the opportunity to start filling that “gender gap” that is still present in our society and in the STEM areas. The road is still long, but creating awareness and promoting diversity at all professional levels is fundamental to push the boundaries, to overcome stereotypes and constrains and to propose new models for the next generations of scientist approaching STEM, driving them to the unique possible direction for an inclusive and sustainable future.

In line with this vision, this Research Topic will help in promoting discussion, breaking the stigma and overcoming existing dogma around Women in Neuroengineering and Neurotechnologies.

